# Supplementary material for: An open-source framework for large-scale, flexible evaluation of biomedical text mining systems
Source: J Biomed Discov Collab. 2008 Jan 29;3:1. doi: 10.1186/1747-5333-3-1 (PMC2276192; doi:10.1186/1747-5333-3-1)
Supplement: Additional file 1 — Evaluation platform source code. The additional file contains the software evaluation framework discussed in this paper. The most current release can be downloaded from SourceForge.net [1]. See the accompanying README file for instructions on installation and use. Also included in the distribution is a collection of UIMA wrappers for some commonly used BioNLP tools and annotated corpora. [file 1747-5333-3-1-S1.zip › bionlp_uima_repository-1.0.1/docs/api/src-html/edu/uchsc/ccp/uima/cc/summary/AnnotationPrinter.html]

```
001    /*
002     *
003     * The Original Code is UCHSC UIMA.
004     *
005     * The Initial Developer of the Original Code is University of Colorado.  
006     * Copyright (C) 2005.  All Rights Reserved.
007     *
008     * UCHSC Utilities was developed by the Center for Computational Pharmacology
009     * (http://compbio.uchsc.edu) at the University of Colorado Health 
010     *  Sciences Center School of Medicine with support from the National 
011     *  Library of Medicine.  
012     *
013     *
014     * Contributor(s):
015     *   William A. Baumgartner <william.baumgartner@uchsc.edu> (Original Author)
016     *   
017     * Created: May 2005
018     * Dependencies:
019     * TODO:
020     *      Various formats should be radio buttons..  not sure if that's possible.
021     *      WordFreak and TnT formats need to be tested, and probably rewritten as they were hacked together to get results for the Corpus grant.
022     *      Output of ClassMention Annotations needs to recursively print the slots
023     *      Default output format assumes that non-syntactic annotations are ClassMention annotations
024     * Changes:
025     *      01/10/06 - wab - Adjusted to conform to updated TextAnnotation class (ClassMentions, SlotMentions, etc.)
026     *      07/04/06 - wab - Added BioCreative format output
027     */
028    package edu.uchsc.ccp.uima.cc.summary;
029    
030    import java.io.IOException;
031    import java.io.PrintStream;
032    import java.util.Iterator;
033    
034    import org.apache.uima.cas.CAS;
035    import org.apache.uima.cas.CASException;
036    import org.apache.uima.collection.CasConsumer_ImplBase;
037    import org.apache.uima.collection.base_cpm.CasObjectProcessor;
038    import org.apache.uima.jcas.JCas;
039    import org.apache.uima.jcas.cas.FSArray;
040    import org.apache.uima.resource.ResourceInitializationException;
041    import org.apache.uima.resource.ResourceProcessException;
042    import org.apache.uima.util.ProcessTrace;
043    
044    import edu.uchsc.ccp.uima.annotation.CCPDocumentInformation;
045    import edu.uchsc.ccp.uima.annotation.CCPDocumentSection;
046    import edu.uchsc.ccp.uima.annotation.CCPTextAnnotation;
047    import edu.uchsc.ccp.uima.annotation.syntactic.CCPClauseAnnotation;
048    import edu.uchsc.ccp.uima.annotation.syntactic.CCPPhraseAnnotation;
049    import edu.uchsc.ccp.uima.annotation.syntactic.CCPTokenAnnotation;
050    import edu.uchsc.ccp.uima.annotation.syntactic.CCPTypedDependency;
051    import edu.uchsc.ccp.uima.util.UIMA_Util;
052    import edu.uchsc.ccp.util.nlp.GenericTextUtils;
053    import edu.uchsc.ccp.util.nlp.annotation.printer.AnnotationPrinterUtil;
054    import edu.uchsc.ccp.util.nlp.annotation.printer.AnnotationToFileOutput;
055    import edu.uchsc.ccp.util.nlp.annotation.printer.DefaultPrintOutput;
056    import edu.uchsc.ccp.util.nlp.annotation.printer.SimpleCollectionReaderFormatOutput;
057    import edu.uchsc.ccp.util.nlp.document.GenericDocument;
058    
059    /**
060     * This class provides a summary of UIMA processing by outputing the annotations in various formats. Some of these formats are still in development,
061     * and may not work well (for instance, TnT format should be used only when every token has a corresponding annotation), however, the main selections
062     * are:<br>
063     * <ul>
064     * <li>To file or To display
065     * <li>WordFreak Format, TnT Format, or default format
066     * </ul>
067     * <br>
068     * The default format should suffice for most uses. The WordFreak and TnT formats were added for the Corpus grant, and have not been fully tested.
069     * <BR>
070     * <BR>
071     * 
072     * 
073     * @author William A. Baumgartner, Jr.
074     * 
075     */
076    public class AnnotationPrinter extends CasConsumer_ImplBase implements CasObjectProcessor {
077        private final boolean DEBUG = false;
078    
079        // private final String indent = " ";
080        //
081        // /**
082        // * this should either be an input parameter or obtained by referencing
083        // * tntOutputAnnotationType.. it was hardcoded as POS to save time
084        // */
085        // private final String printConceptMentionSlotForType = "POS";
086    
087        /**
088         * A boolean flag represented by "OutputToFile" in the descriptor file. If true, the program outputs to the file specified by "OutputFileName,"
089         * otherwise, output is directed to the display.
090         */
091        public final String PARAM_OUTPUTTOFILE = "OutputToFile";
092    
093        /**
094         * The path to the output file must be included in the descriptor under "OutputFileName"
095         */
096        public final String PARAM_OUTPUTFILENAME = "OutputFileName";
097    
098        /**
099         * A boolean flag represented by "UseTnTFormat" in the descriptor file. If true, the program outputs using an embedded format, e.g. token1/tag1
100         * token2/tag2 ...
101         */
102        public final String PARAM_USE_BRILLFORMAT = "UseBrillFormat";
103    
104        /**
105         * A boolean flag represented by "UseWordFreakFormat" in the descriptor file. If true, the program outputs using an XML format similar to
106         * WordFreak.
107         */
108        public final String PARAM_USE_WORDFREAKFORMAT = "UseWordFreakFormat";
109    
110        public final String PARAM_USE_EMBEDDEDXMLFORMAT = "UseEmbeddedXMLFormat";
111    
112        public final String PARAM_USE_BIOCREATIVEFORMAT = "UseBiocreativeFormat";
113    
114        public final String PARAM_USE_BIOCREATIVENORMFORMAT = "UseBiocreativeNormFormat";
115    
116        public final String PARAM_OUTPUT_INTO_MULTIPLE_FILES = "OutputInToMultipleFiles";
117    
118        public final String PARAM_USE_ONELINE_FORMAT = "UseOneLineOutputFormat";
119    
120        public final String PARAM_USE_MUTATION_OUTPUT_FORMAT = "UseMutationOutputFormat";
121    
122        public final String PARAM_USE_THING_WITH_ENTREZ_ID_FORMAT = "UseThingWithEntrezIDFormat";
123    
124        public final String PARAM_USE_ALT_EVAL_FORMAT = "UseAltEvalFormat";
125        
126        public final String PARAM_USE_SIMPLE_COLLECTION_READER_FORMAT = "UseSimpleCollectionReaderFormat";
127        
128        public final String PARAM_USE_ANNOTATION_TO_FILE_FORMAT = "UseAnnotationToFileFormat";
129    
130        // /**
131        // * A string represented by "AnnotationTypeToOutput" in the descriptor file.
132        // * This string can either be set to "ALL" to have all annotations printed,
133        // * or to a specific annotation type, e.g.
134        // * "edu.uchsc.ccp.uima.annotation.TokenAnnotation" to limit the annotations
135        // * outputted to a single type.
136        // */
137        // public final String PARAM_OUTPUTANNOTATIONTYPE = "AnnotationTypeToOutput";
138    
139        private boolean outputToFile = false;
140    
141        private boolean useBrillFormat = false;
142    
143        private boolean useWordFreakFormat = false;
144    
145        private boolean useEmbeddedXMLFormat = false;
146    
147        private boolean useBioCreativeFormat = false;
148    
149        private boolean useBioCreativeNormFormat = false;
150    
151        private boolean outputIntoMultipleFiles = false;
152    
153        private boolean useMutationOutputFormat = false;
154    
155        private boolean useThingWithEntrezIDFormat = false;
156    
157        private boolean useAltEvalFormat = false;
158        private boolean useSimpleCollectionReaderFormat = false;
159    
160        private boolean useOneLineFormat = false;
161    
162        private String outputFileName = "";
163    
164    //    private AnnotatorValidator annotatorValidator;
165    //
166    //    private AnnotationSetValidator annotationSetValidator;
167    
168        // private String annotationTypeToOutput = "";
169    
170        private PrintStream annotationPrinterStream = System.out;
171    
172        private AnnotationPrinterUtil annotationPrinterUtil;
173    
174        /**
175         * Initializes the CAS Consumer by reading in parameters from the descriptor file.
176         */
177        public void initialize() throws ResourceInitializationException {
178    
179            outputToFile = ((Boolean) getConfigParameterValue(PARAM_OUTPUTTOFILE)).booleanValue();
180    //        useBrillFormat = ((Boolean) getConfigParameterValue(PARAM_USE_BRILLFORMAT)).booleanValue();
181    //        useWordFreakFormat = ((Boolean) getConfigParameterValue(PARAM_USE_WORDFREAKFORMAT)).booleanValue();
182    //        useEmbeddedXMLFormat = ((Boolean) getConfigParameterValue(PARAM_USE_EMBEDDEDXMLFORMAT)).booleanValue();
183    //        useBioCreativeFormat = ((Boolean) getConfigParameterValue(PARAM_USE_BIOCREATIVEFORMAT)).booleanValue();
184    //        useBioCreativeNormFormat = ((Boolean) getConfigParameterValue(PARAM_USE_BIOCREATIVENORMFORMAT)).booleanValue();
185    //        outputIntoMultipleFiles = ((Boolean) getConfigParameterValue(PARAM_OUTPUT_INTO_MULTIPLE_FILES)).booleanValue();
186            useOneLineFormat = ((Boolean) getConfigParameterValue(PARAM_USE_ONELINE_FORMAT)).booleanValue();
187    //        useMutationOutputFormat = ((Boolean) getConfigParameterValue(PARAM_USE_MUTATION_OUTPUT_FORMAT)).booleanValue();
188    //        useThingWithEntrezIDFormat = ((Boolean) getConfigParameterValue(PARAM_USE_THING_WITH_ENTREZ_ID_FORMAT)).booleanValue();
189    //        useAltEvalFormat = ((Boolean) getConfigParameterValue(PARAM_USE_ALT_EVAL_FORMAT)).booleanValue();
190            useSimpleCollectionReaderFormat = ((Boolean) getConfigParameterValue(PARAM_USE_SIMPLE_COLLECTION_READER_FORMAT)).booleanValue();
191            boolean useAnnotationFileOutput = ((Boolean) getConfigParameterValue(PARAM_USE_ANNOTATION_TO_FILE_FORMAT)).booleanValue();
192            
193            
194            // get the output file name
195            outputFileName = (String) getConfigParameterValue(PARAM_OUTPUTFILENAME);
196            // annotationTypeToOutput = (String) getConfigParameterValue(PARAM_OUTPUTANNOTATIONTYPE);
197    
198            if (DEBUG) {
199                System.out.println("----- Annotation Printer Input Parameters -----");
200                System.out.println("-- outputToFile = " + outputToFile);
201                System.out.println("-- outputFileName = " + outputFileName);
202                System.out.println("-- useTnTFormat = " + useBrillFormat);
203                System.out.println("-- useWordFreakFormat = " + useWordFreakFormat);
204                System.out.println("-- useEmbeddedXMLFormat = " + useEmbeddedXMLFormat);
205                // System.out.println("-- tntOutputAnnotationType = " + annotationTypeToOutput);
206            }
207    
208            if (outputToFile) {
209                try {
210                    annotationPrinterStream = GenericTextUtils.openPrintStream(outputFileName);
211                } catch (IOException ioe) {
212                    System.err.println("Error creating annotation printer printstream.");
213                    ioe.printStackTrace();
214                }
215            } else { // just print to the screen
216                annotationPrinterStream = System.out;
217            }
218    
219    //        if (useBrillFormat) {
220    //            annotationPrinterUtil = new BrillOutput();
221    //        } else if (useWordFreakFormat) {
222    //            annotationPrinterUtil = new WordFreakOutput();
223    //        } else if (useEmbeddedXMLFormat) {
224    //            annotationPrinterUtil = new EmbeddedXMLOutput();
225    //            ((EmbeddedXMLOutput) annotationPrinterUtil).printEmbeddedXMLHeader(annotationPrinterStream);
226    //        } else if (useMutationOutputFormat) {
227    //            annotationPrinterUtil = new MutationAnnotationOutput();
228    //        } else if (useBioCreativeFormat) {
229    //            annotationPrinterUtil = new BioCreativeOutput();
230    //        } else if (useBioCreativeNormFormat) {
231    //            annotationPrinterUtil = new BioCreativeNormOutput();
232    //        } else if (useThingWithEntrezIDFormat) {
233    //            annotationPrinterUtil = new ThingsWithEntrezIDOutput();
234    //        } else if (useAltEvalFormat) {
235    //            annotationPrinterUtil = new AltEvalFormatOutput();
236    //        } else 
237                if (useSimpleCollectionReaderFormat) {
238                annotationPrinterUtil = new SimpleCollectionReaderFormatOutput();
239            } else if (useAnnotationFileOutput) {
240                annotationPrinterUtil = new AnnotationToFileOutput();
241            } else {
242                // use default output format
243                annotationPrinterUtil = new DefaultPrintOutput();
244                ((DefaultPrintOutput) annotationPrinterUtil).setUseOneLineFormatStatus(useOneLineFormat);
245            }
246    
247            super.initialize();
248        }
249    
250        /*
251         * (non-Javadoc)
252         * 
253         * @see com.ibm.uima.collection.base_cpm.CasObjectProcessor#processCas(com.ibm.uima.cas.CAS)
254         */
255        /**
256         * Prints out the CAS annotations in the format specified by the descriptor file.
257         */
258        public void processCas(CAS aCAS) throws ResourceProcessException {
259            JCas jcas;
260            try {
261                jcas = aCAS.getJCas();
262            } catch (CASException e) {
263                System.out.println("Exception getting CAS in AnnotationPrinter");
264                e.printStackTrace();
265                throw new ResourceProcessException(e);
266            }
267    
268            GenericDocument gd = new GenericDocument();
269            if (DEBUG) {
270                System.err.println("DEBUG -- AnnotationPrinter: About to add document to JCas");
271                gd.printDocument(System.err);
272            }
273    
274            UIMA_Util.swapDocumentInfo(jcas, gd);
275    
276            if (DEBUG) {
277                System.err.println("# annotations in GD: " + gd.getAnnotations().size());
278            }
279    
280            annotationPrinterUtil.printDocument(gd, annotationPrinterStream);
281    
282        }
283    
284        // Iterator docInfoIter = jcas.getJFSIndexRepository().getAnnotationIndex(CCPDocumentInformation.type).iterator();
285        // Iterator docSectionIter = jcas.getJFSIndexRepository().getAnnotationIndex(CCPDocumentSection.type).iterator();
286        // String docText = jcas.getDocumentText();
287    
288        // if (useTnTFormat) { // output annotations in TnT/Brill format
289        // try {
290        // Class c = Class.forName(annotationTypeToOutput);
291        // Field f = c.getField("type");
292        // if (DEBUG) {
293        // System.out.println("FIELD: " + f.toString() + "\tVALUE: " +
294        // f.getInt(c));
295        // }
296        // Iterator annotationIter =
297        // jcas.getJFSIndexRepository().getAnnotationIndex(f.getInt(c)).iterator();
298        // tntOutput(docInfoIter, docSectionIter, annotationIter, docText);
299        // } catch (Exception e) {
300        // System.err.println("Exception while getting annotations for
301        // AnnotationPrinter TnT output format...");
302        // e.printStackTrace();
303        // }
304        // } else if (useWordFreakFormat) {
305        //
306        // Iterator annotationIter =
307        // jcas.getJFSIndexRepository().getAnnotationIndex(CCPTextAnnotation.type).iterator();
308        // wordFreakOutput(docInfoIter, docSectionIter, annotationIter,
309        // docText);
310        //
311        // } else { // use default format, this outputs all annotations
312        // Iterator annotationIter;
313        // if (annotationTypeToOutput.equalsIgnoreCase("ALL")) {
314        // // annotationIter =
315        // // jcas.getJFSIndexRepository().getAnnotationIndex(DefaultAnnotation.type).iterator();
316        // annotationIter = jcas.getJFSIndexRepository().getAnnotationIndex(CCPTextAnnotation.type).iterator();
317        // } else {
318        // try {
319        // Class c = Class.forName(annotationTypeToOutput);
320        // Field f = c.getField("type");
321        // annotationIter = jcas.getJFSIndexRepository().getAnnotationIndex(f.getInt(c)).iterator();
322        // } catch (Exception e) {
323        // System.err.println("Possible invalid annotation type selected for output in AnnotationPrinter...");
324        // e.printStackTrace();
325        // annotationIter = null;
326        // }
327        // }
328        //        
329        //        
330        // defaultOutput(docInfoIter, docSectionIter, annotationIter, docText);
331    
332        // }
333    
334        // }
335    
336        // private void wordFreakOutput(Iterator docInfoIter, Iterator
337        // docSectionIter, Iterator annotationIter, String docText) {
338        // annotationPrinterStream.println("The WordFreak output is currently
339        // unavailable... it needs to be rewritten to comply with the new
340        // TypeSystem.");
341        // annotationPrinterStream.println("<?xml version=\"1.0\"
342        // encoding=\"UTF-8\"?>");
343        //
344        // // print document information
345        // String docID = "-1";
346        // int docSize = -1;
347        // if (docInfoIter.hasNext()) {
348        // CCPDocumentInformation docInfo = (CCPDocumentInformation)
349        // docInfoIter.next();
350        // docID = docInfo.getDocumentID();
351        // docSize = docInfo.getDocumentSize();
352        // }
353        // annotationPrinterStream.println("DOCUMENT ID: " + docID);
354        //        
355        // // print document section information
356        // String sections = "";
357        // while (docSectionIter.hasNext()) {
358        // CCPDocumentSection docSection = (CCPDocumentSection)
359        // docSectionIter.next();
360        // int sectionID = docSection.getSectionID();
361        // int sectionLength = docSection.getSectionLength();
362        // String section = "Other";
363        // if (sectionID == 1) {
364        // section = "Title";
365        // } else if (sectionID == 2) {
366        // section = "Abstract";
367        // } else if (sectionID == 3) {
368        // section = "Other Abstract";
369        // }
370        // sections += (section + "(" + sectionLength + ") ");
371        // }
372        // annotationPrinterStream.println("DOCUMENT LENGTH: " + docSize);
373        // annotationPrinterStream.println("DOCUMENT SECTIONS: " + sections);
374        // annotationPrinterStream.println("DOCUMENT: " + docText);
375        //
376        // int numIndents = 0;
377        // annotationPrinterStream.println("<AnnotationFile version=\"1.0\">");
378        // numIndents++;
379        // printIndents(numIndents);
380        // // G //annotationPrinterStream.println("<features
381        // // list=\"location,annotator,id\"/>");
382        // annotationPrinterStream.println("<features
383        // list=\"filename,annotator,id\"/>");
384        // printIndents(numIndents);
385        // // G //annotationPrinterStream.println("<annotators list=\"GENIA\"/>");
386        // annotationPrinterStream.println("<annotators
387        // list=\"Blaschke,KeX,TnT\"/>");
388        // printIndents(numIndents);
389        // // G //annotationPrinterStream.println("<Annotation type=\"document\"
390        // // span=0.." + docText.length() +" confidence=\"1.0\"
391        // // location=\"DatabaseTable: Genia_corpus\" annotator=\"GENIA\" id=" +
392        // // docID + "\">");
393        // annotationPrinterStream.println("<Annotation type=\"Document\" span=0.."
394        // + docText.length()
395        // + " confidence=\"1.0\" location=\"/data/blaschke_corpus.dat\"
396        // annotator=\"Blaschke\" id=" + docID + "\">");
397        // numIndents++;
398        //
399        // // sort annotations by start of span
400        // HashMap sortedIDsHash = new HashMap();
401        // HashMap annotationsHash = new HashMap();
402        //
403        // int count = 0;
404        // while (annotationIter.hasNext()) {
405        // CCPTextAnnotation annot = (CCPTextAnnotation) annotationIter.next();
406        // TextAnnotation ta = annot.convertToTextAnnotation(); // new
407        // // TextAnnotation(annot);
408        // ta.setAnnotationID(count); // it is possible that the annotation id
409        // // has not yet been set, so we reset
410        // // them here.. this should not affect
411        // // anything outside of this program
412        //
413        // if (!doingBlaschke) {
414        // annotationsHash.put(Integer.toString(ta.getAnnotationID()), ta);
415        // sortedIDsHash.put(Integer.toString(ta.getAnnotationID()), new
416        // Integer(ta.getBeginIndex()));
417        // } else {
418        // // don't print gene annotations from tnt or tokens from sentence
419        // // tokenizer
420        // if (!((ta.getConceptMentionTypes()[0]).equals("Gene") |
421        // (ta.getConceptMentionTypes()[0]).equals("Token"))) {
422        // // change all NEWGENEs to NNs
423        // if ((ta.getConceptMentionTypes()[0]).equals("POS") &
424        // (ta.getConceptMentionSlotValues()[0]).startsWith("NEWGENE")) {
425        // ta.setConceptMentionSlotValueForType("POS", "NN");
426        // }
427        // annotationsHash.put(Integer.toString(ta.getAnnotationID()), ta);
428        // sortedIDsHash.put(Integer.toString(ta.getAnnotationID()), new
429        // Integer(ta.getBeginIndex()));
430        // }
431        // }
432        //
433        // count++;
434        // }
435        //
436        // // System.err.println("Sorting IDs by span beginning...");
437        // ArrayList sortedIDs =
438        // GenericTextUtils.sortHashMapByValues(sortedIDsHash);
439        //
440        // // System.err.println("Printing ordered annotations... count = " +
441        // // sortedIDs.size());
442        // ArrayList sortedAnnotations = new ArrayList(); // will eventually be
443        // // sorted first by
444        // // increasing span
445        // // begin, then by
446        // // decreasing span end
447        // // int lastStart = -1;
448        // // int lastEnd = 999999999;
449        //
450        // // if (DEBUG) {
451        // //
452        // System.out.println("=====================================================================================");
453        // // System.out.println("=============================== SORTED BY SPAN
454        // // START ===============================");
455        // //
456        // System.out.println("=====================================================================================");
457        // // }
458        // for (int i = sortedIDs.size() - 1; i > -1; i--) {
459        // KeyValuePair kvp = (KeyValuePair) sortedIDs.get(i);
460        // // int start = kvp.getValue();
461        // TextAnnotation annot = (TextAnnotation)
462        // annotationsHash.get(kvp.getKey());
463        // // System.out.println(getWordFreakAnnotationString(annot));
464        // addAnnotationToSortedList(sortedAnnotations, annot);
465        // }
466        //
467        // if (DEBUG) {
468        // System.out.println("*************************************************************************************");
469        // System.out.println("======================= SORTED BY SPAN START, THEN
470        // END =============================");
471        // System.out.println("*************************************************************************************");
472        //
473        // for (int i = 0; i < sortedAnnotations.size(); i++) {
474        // TextAnnotation ta = (TextAnnotation) sortedAnnotations.get(i);
475        // System.out.println(getWordFreakAnnotationString(ta));
476        // }
477        // }
478        //
479        // if (DEBUG) {
480        // System.out.println("+++++++++++++++++++++++++++++++++++++++++++++++++++++++++++++++++++++++++++++++++++++");
481        // System.out.println("================================== SORTED AND
482        // INDENTED =============================");
483        // System.out.println("+++++++++++++++++++++++++++++++++++++++++++++++++++++++++++++++++++++++++++++++++++++");
484        // }
485        //
486        // Stack endSpansStack = new Stack();
487        //
488        // // we must add two filler annotations to represent the ends to the
489        // // <AnnotationFile and the <Annotation type=document from above
490        // endSpansStack.push(new Integer(docText.length() + 1));
491        // endSpansStack.push(new Integer(docText.length()));
492        // for (int i = 0; i < sortedAnnotations.size(); i++) {
493        // TextAnnotation ta = (TextAnnotation) sortedAnnotations.get(i);
494        //
495        // // check to see if a previous annotation needs to be closed
496        // Integer topEndSpan = (Integer) endSpansStack.peek();
497        // while (ta.getEndIndex() > topEndSpan.intValue()) { // pop the
498        // // topEndSpan,
499        // // close
500        // // annotation
501        // numIndents--;
502        // printCloseAnnotation(numIndents);
503        // endSpansStack.pop();
504        // topEndSpan = (Integer) endSpansStack.peek();
505        // }
506        //
507        // if (getIndentRank(ta.getConceptMentionTypes()[0]) > 0) {
508        // // push the end of the span onto the stack
509        // endSpansStack.push(new Integer(ta.getEndIndex()));
510        // printOpenAnnotation(numIndents, ta, false);
511        // numIndents++;
512        // } else {
513        // printOpenAnnotation(numIndents, ta, true);
514        // }
515        //
516        // }
517        //
518        // // pop elements off the stack and close annotations
519        // Enumeration enumer = endSpansStack.elements();
520        // while (enumer.hasMoreElements()) {
521        // numIndents--;
522        // printCloseAnnotation(numIndents);
523        // endSpansStack.pop();
524        // }
525        //
526        // }
527        //
528        // private void printCloseAnnotation(int numIndents) {
529        // for (int i = 0; i < numIndents; i++) {
530        // annotationPrinterStream.print(indent);
531        // }
532        // annotationPrinterStream.println("</Annotation>");
533        //
534        // }
535        //
536        // private void printOpenAnnotation(int numIndents, TextAnnotation ta,
537        // boolean isClosed) {
538        // printIndents(numIndents);
539        // if (isClosed) {
540        // annotationPrinterStream.println(getWordFreakAnnotationString(ta) + "/>");
541        // } else {
542        // annotationPrinterStream.println(getWordFreakAnnotationString(ta) + ">");
543        // }
544        // }
545        //
546        // private void printIndents(int numIndents) {
547        // for (int i = 0; i < numIndents; i++) {
548        // annotationPrinterStream.print(indent);
549        // }
550        // }
551        //
552        //
553        // private void addAnnotationToSortedList(ArrayList sortedAnnotations,
554        // TextAnnotation ta) {
555        // final int debugLength = 20;
556        // if (sortedAnnotations.size() == 0) { // if the sorted list is empty,
557        // // then add the first annotation
558        // sortedAnnotations.add(ta);
559        // } else {
560        // // traverse down list until span start is >= to a span start that is
561        // // found
562        // int spanStart = ta.getBeginIndex();
563        // int spanEnd = ta.getEndIndex();
564        // String cmType = ta.getConceptMentionTypes()[0];
565        // int indentRank = getIndentRank(cmType);
566        // int index = 0;
567        // while (index < sortedAnnotations.size() && spanStart > ((TextAnnotation)
568        // sortedAnnotations.get(index)).getBeginIndex()) {
569        // index++;
570        // }
571        //
572        // if (index == sortedAnnotations.size()) { // then there are no
573        // // annotations in the
574        // // sorted list with the
575        // // same or less span
576        // // start, so add this
577        // // annotation to the end
578        // if (DEBUG & sortedAnnotations.size() < debugLength) {
579        // System.out.println("------------------------ Adding to end 1");
580        // }
581        // sortedAnnotations.add(ta);
582        // } else { // we are not at the end of the list, so we must sort
583        // // the new ta based on the span end
584        // // if span end is greater than the span end of the stored ta,
585        // // then insert the new ta before it
586        //
587        // while (index < sortedAnnotations.size()
588        // && (spanStart == ((TextAnnotation)
589        // sortedAnnotations.get(index)).getBeginIndex() & spanEnd <
590        // ((TextAnnotation) sortedAnnotations
591        // .get(index)).getEndIndex())) {
592        // index++;
593        // }
594        // if (index == sortedAnnotations.size()) { // then there are no
595        // // annotations in
596        // // the sorted list
597        // // with the same or
598        // // greater span end,
599        // // so add this
600        // // annotation to the
601        // // end
602        // if (DEBUG & sortedAnnotations.size() < debugLength) {
603        // System.out.println("-------------------------- Adding to end 2");
604        // }
605        // sortedAnnotations.add(ta);
606        // } else {
607        //
608        // // if you have annotations with the same spans...
609        // if (spanStart == ((TextAnnotation)
610        // sortedAnnotations.get(index)).getBeginIndex()
611        // & spanEnd == ((TextAnnotation)
612        // sortedAnnotations.get(index)).getEndIndex()) {
613        // // now sort down based on indentRank
614        // while (index < sortedAnnotations.size()
615        // && (spanStart == ((TextAnnotation)
616        // sortedAnnotations.get(index)).getBeginIndex()
617        // & spanEnd == ((TextAnnotation)
618        // sortedAnnotations.get(index)).getEndIndex() & indentRank <
619        // getIndentRank(((TextAnnotation) sortedAnnotations
620        // .get(index)).getConceptMentionTypes()[0]))) {
621        // index++;
622        // }
623        //
624        // if (index == sortedAnnotations.size()) { // then
625        // // there are
626        // // no
627        // // annotations
628        // // in the
629        // // sorted
630        // // list with
631        // // the same
632        // // or
633        // // greater
634        // // span end,
635        // // so add
636        // // this
637        // // annotation
638        // // to the
639        // // end
640        // if (indentRank > getIndentRank(((TextAnnotation)
641        // sortedAnnotations.get(index - 1)).getConceptMentionTypes()[0])) {
642        // if (DEBUG & sortedAnnotations.size() < debugLength) {
643        // System.out.println("-------------------------- Adding in front 1");
644        // }
645        // sortedAnnotations.add(index - 1, ta);
646        // } else {
647        // if (DEBUG & sortedAnnotations.size() < debugLength) {
648        // System.out.println("-------------------------- Adding to end 3");
649        // }
650        // sortedAnnotations.add(ta);
651        // }
652        // } else {
653        // if (indentRank > getIndentRank(((TextAnnotation)
654        // sortedAnnotations.get(index)).getConceptMentionTypes()[0])) {
655        // if (DEBUG & sortedAnnotations.size() < debugLength) {
656        // System.out.println("-------------------------- Adding in front 2");
657        // }
658        // sortedAnnotations.add(index, ta);
659        // } else {
660        // if (DEBUG & sortedAnnotations.size() < debugLength) {
661        // System.out.println("-------------------------- Adding in back 4");
662        // }
663        // sortedAnnotations.add(index + 1, ta);
664        // }
665        // //
666        // // if (DEBUG & sortedAnnotations.size() <
667        // // debugLength) {
668        // // System.out.println("---------------------------
669        // // Adding in front: index = " + index);
670        // // }
671        // // sortedAnnotations.add(index, ta);
672        // // // // if you've found the right place to add
673        // // this, then
674        // // if (indexRank >
675        // //
676        // getIndentRank(((TextAnnotation)sortedAnnotations.get(index)).getConceptMentionTypes()[0]))
677        // // {
678        // // sortedAnnotations.add(index, ta);
679        // // } else {
680        // // sortedAnnotations.add(index+1, ta);
681        // // }
682        // }
683        // } else { // we don't need to worry about rank because the
684        // // spans are not equal, so just add it
685        // if (DEBUG & sortedAnnotations.size() < debugLength) {
686        // System.out.println("-------------------------- Adding in front 3");
687        // }
688        // sortedAnnotations.add(index, ta);
689        // }
690        // }
691        // }
692        // }
693        //
694        // if (DEBUG) {
695        // if (sortedAnnotations.size() < debugLength) {
696        // System.out.println("++++ ADDED ANNOTATION TO SORTED LIST:");
697        // for (int i = 0; i < sortedAnnotations.size(); i++) {
698        // TextAnnotation annot = (TextAnnotation) sortedAnnotations.get(i);
699        // System.out.println(getWordFreakAnnotationString(annot));
700        // }
701        // }
702        // }
703        //
704        // }
705        //
706        // /**
707        // * Returns a rank to tell if one annotition should be "inside" another
708        // *
709        // * @param type
710        // * @return
711        // */
712        // private int getIndentRank(String type) {
713        // int indentRank = 0;
714        //
715        // if (type.equals("POS")) {
716        // indentRank = 0;
717        // } else if (type.equals("Sentence")) {
718        // indentRank = 4;
719        // } else if (type.equals("DocumentSection")) {
720        // indentRank = 5;
721        // } else {
722        // indentRank = 2;
723        // }
724        //
725        // return indentRank;
726        // }
727        //
728        // private String getWordFreakAnnotationString(TextAnnotation ta) {
729        //
730        // String annotatorName = "";
731        // if (ta.getAnnotatorID() == 1) {
732        // annotatorName = "TnT";
733        // } else if (ta.getAnnotatorID() == 2) {
734        // annotatorName = "ABNER";
735        // } else if (ta.getAnnotatorID() == 3) {
736        // annotatorName = "KeX";
737        // } else if (ta.getAnnotatorID() == 4) {
738        // annotatorName = "GENIA";
739        // } else if (ta.getAnnotatorID() == 5) {
740        // annotatorName = "AbGene";
741        // } else if (ta.getAnnotatorID() == 12) {
742        // annotatorName = "Blaschke";
743        // } else {
744        // annotatorName = "tagger";
745        // }
746        //
747        // String docSection;
748        // if (ta.getDocumentSectionID() == 1) {
749        // docSection = "Title";
750        // } else if (ta.getDocumentSectionID() == 2) {
751        // docSection = "Abstract";
752        // } else {
753        // docSection = "DocumentSection";
754        // }
755        //
756        // String cmType = ta.getConceptMentionTypes()[0];
757        // String s;
758        // if (cmType.equals("POS")) {
759        // s = "<Annotation type=\"" + ta.getConceptMentionSlotValues()[0] + "\"
760        // span=\"" + Integer.toString(ta.getBeginIndex()) + ".."
761        // + Integer.toString(ta.getEndIndex()) + "\" confidence=\"1.0\"
762        // annotator=\"" + annotatorName + "\" id=\""
763        // + ta.getAnnotationID() + "\"";// + " indentRank: " +
764        // // getIndentRank(ta.getConceptMentionTypes()[0]);
765        // } else if (cmType.equals("DocumentSection")) {
766        // s = "<Annotation type=\"" + docSection + "\" span=\"" +
767        // Integer.toString(ta.getBeginIndex()) + ".."
768        // + Integer.toString(ta.getEndIndex()) + "\" confidence=\"1.0\"
769        // annotator=\"" + annotatorName + "\" id=\""
770        // + ta.getAnnotationID() + "\"";// + " indentRank: " +
771        // // getIndentRank(ta.getConceptMentionTypes()[0]);
772        // } else {
773        // s = "<Annotation type=\"" + ta.getConceptMentionTypes()[0] + "\" span=\""
774        // + Integer.toString(ta.getBeginIndex()) + ".."
775        // + Integer.toString(ta.getEndIndex()) + "\" confidence=\"1.0\"
776        // annotator=\"" + annotatorName + "\" id=\""
777        // + ta.getAnnotationID() + "\"";// + " indentRank: " +
778        // // getIndentRank(ta.getConceptMentionTypes()[0]);
779        // }
780        // return s;
781        // }
782        //
783        // private void tntOutput(Iterator docInfoIter, Iterator docSectionIter,
784        // Iterator annotationIter, String docText) {
785        // annotationPrinterStream.println("The WordFreak output is currently
786        // unavailable... it needs to be rewritten to comply with the new
787        // TypeSystem.");
788        // // print document information
789        // String docID = "-1";
790        // int docSize = -1;
791        // if (docInfoIter.hasNext()) {
792        // CCPDocumentInformation docInfo = (CCPDocumentInformation)
793        // docInfoIter.next();
794        // docID = docInfo.getDocumentID();
795        // docSize = docInfo.getDocumentSize();
796        // }
797        // annotationPrinterStream.println("DOCUMENT ID: " + docID);
798        //        
799        // // print document section information
800        // String sections = "";
801        // while (docSectionIter.hasNext()) {
802        // CCPDocumentSection docSection = (CCPDocumentSection)
803        // docSectionIter.next();
804        // int sectionID = docSection.getSectionID();
805        // int sectionLength = docSection.getSectionLength();
806        // String section = "Other";
807        // if (sectionID == 1) {
808        // section = "Title";
809        // } else if (sectionID == 2) {
810        // section = "Abstract";
811        // } else if (sectionID == 3) {
812        // section = "Other Abstract";
813        // }
814        // sections += (section + "(" + sectionLength + ") ");
815        // }
816        // annotationPrinterStream.println("DOCUMENT LENGTH: " + docSize);
817        // annotationPrinterStream.println("DOCUMENT SECTIONS: " + sections);
818        // annotationPrinterStream.println("DOCUMENT: " + docText);
819        //
820        // // iterate over annotations, save them in sorted order
821        // // this is a bit of a hack... keyvaluepair should be rewritten to be
822        // // able to hold objects, not just strings
823        // HashMap annotationsHash = new HashMap();
824        // HashMap hashToSort = new HashMap();
825        //
826        // int count = 0;
827        // while (annotationIter.hasNext()) {
828        // DefaultAnnotation annot = (DefaultAnnotation) annotationIter.next();
829        // TextAnnotation ta = annot.convertToTextAnnotation(); // new
830        // // TextAnnotation(annot);
831        // ta.setAnnotationID(count); // it is possible that the annotation id
832        // // has not yet been set, so we reset
833        // // them here.. this should not affect
834        // // anything outside of this program
835        // annotationsHash.put(Integer.toString(ta.getAnnotationID()), ta);
836        // hashToSort.put(Integer.toString(ta.getAnnotationID()), new
837        // Integer(ta.getEndIndex()));
838        // count++;
839        // }
840        //
841        // ArrayList sortedAnnotationIDs =
842        // GenericTextUtils.sortHashMapByValues(hashToSort);
843        //
844        // if (DEBUG) {
845        // System.out.println("************ There are " + count + " tnt annotations
846        // for this document. ************");
847        // System.out.println("************ There are " + hashToSort.size() + "
848        // unsorted annotations for this document. ************");
849        // System.out.println("************ There are " + sortedAnnotationIDs.size()
850        // + " sorted annotations for this document. ************");
851        // }
852        //
853        // String annotationText;
854        // // for (int i = sortedAnnotationIDs.size()-1; i > 270; i--) { // for
855        // // each sorted annotationid, get annotation, add to text
856        // for (int i = 0; i < sortedAnnotationIDs.size(); i++) { // for each
857        // // sorted
858        // // annotationid,
859        // // get
860        // // annotation,
861        // // add to text
862        // // do in reverse order so that spans are not changed from the
863        // // beginning
864        // KeyValuePair kvp = (KeyValuePair) sortedAnnotationIDs.get(i);
865        // String annotationID = kvp.getKey();
866        // int insertIndex = kvp.getValue();
867        //
868        // TextAnnotation ta = (TextAnnotation) annotationsHash.get(annotationID);
869        // annotationText =
870        // ta.getConceptMentionSlotValueForType(printConceptMentionSlotForType);
871        //
872        // String space = "";
873        // if (insertIndex != docText.length()) { // is not the final tag,
874        // // then check to see if a
875        // // space needs to be added
876        // // after the tag
877        // if (docText.charAt(insertIndex) == ' ') {
878        // space = "";
879        // } else {
880        // space = " ";
881        // }
882        // }
883        //
884        // docText = docText.substring(0, insertIndex) + "/" + annotationText +
885        // space + docText.substring(insertIndex);
886        //
887        // if (DEBUG) {
888        // // ta.printAnnotation(System.out);
889        // // System.out.println(docText);
890        // }
891        // }
892        //
893        // // add the docid to the beginning
894        // docText = "@@" + docID + " " + docText;
895        //
896        // // print the tnt formatted doctext
897        // annotationPrinterStream.println(docText);
898        //
899        // }
900    
901        /*
902         * The default output for the AnnotationPrinter
903         * 
904         * @param sourceDocInfoIter @param annotationIter @param docText
905         */
906    //    private void defaultOutput(Iterator docInfoIter, Iterator docSectionIter, Iterator annotationIter, String docText) {
907    //        annotationPrinterStream.println("==============================================================================");
908    //        // print document information
909    //        String docID = "-1";
910    //        int docSize = -1;
911    //        if (docInfoIter.hasNext()) {
912    //            CCPDocumentInformation docInfo = (CCPDocumentInformation) docInfoIter.next();
913    //            docID = docInfo.getDocumentID();
914    //            docSize = docInfo.getDocumentSize();
915    //        }
916    //        annotationPrinterStream.println("DOCUMENT ID: " + docID);
917    //
918    //        // print document section information
919    //        String sections = "";
920    //        while (docSectionIter.hasNext()) {
921    //            CCPDocumentSection docSection = (CCPDocumentSection) docSectionIter.next();
922    //            int sectionID = docSection.getSectionID();
923    //            int sectionLength = docSection.getSectionLength();
924    //            String section = "Other";
925    //            if (sectionID == 1) {
926    //                section = "Title";
927    //            } else if (sectionID == 2) {
928    //                section = "Abstract";
929    //            } else if (sectionID == 3) {
930    //                section = "Other Abstract";
931    //            }
932    //            sections += (section + "(" + sectionLength + ")  ");
933    //        }
934    //        annotationPrinterStream.println("DOCUMENT LENGTH: " + docSize);
935    //        annotationPrinterStream.println("DOCUMENT SECTIONS: " + sections);
936    //        annotationPrinterStream.println("DOCUMENT: " + docText);
937    //
938    //        // iterate and print annotations
939    //        // annotationPrinterStream.println("------------------------------------------------------------------------------------");
940    //        // annotationPrinterStream.println("------------------------------ ANNOTATIONS -------------------------------");
941    //        // annotationPrinterStream.println("------------------------------------------------------------------------------------");
942    //        while (annotationIter.hasNext()) {
943    //            CCPTextAnnotation annot = (CCPTextAnnotation) annotationIter.next();
944    //            String coveredText = annot.getCoveredText();
945    //            int beginIndex = annot.getBegin();
946    //            int endIndex = annot.getEnd();
947    //            int docSection;
948    //            String section;
949    //            if (Integer.toString(annot.getDocumentSectionID()) != null) {
950    //                docSection = annot.getDocumentSectionID();
951    //                section = "Other";
952    //                if (docSection == 1) {
953    //                    section = "Title";
954    //                } else if (docSection == 2) {
955    //                    section = "Abstract";
956    //                } else if (docSection == 3) {
957    //                    section = "Other Abstract";
958    //                }
959    //            } else {
960    //                docSection = 0;
961    //                section = "No section";
962    //            }
963    //
964    //            String abbreviatedType = annot.getType().getName().substring(annot.getType().getName().lastIndexOf(".") + 1);
965    //            if (annot.getType().getName().equals("edu.uchsc.ccp.uima.annotation.syntactic.TokenAnnotation")) {
966    //                FSArray tdArray = ((CCPTokenAnnotation) annot).getTypedDependencies();
967    //                String typedDependenciesStr = "";
968    //                if (tdArray != null) {
969    //                    for (int i = 0; i < tdArray.size(); i++) {
970    //                        CCPTypedDependency ccpTD = (CCPTypedDependency) tdArray.get(i);
971    //                        typedDependenciesStr += ("[GOV: " + ccpTD.getGovernorTokenNum());
972    //                        typedDependenciesStr += (" DEP: " + ccpTD.getDependentTokenNum());
973    //                        typedDependenciesStr += (" REL: " + ccpTD.getRelation() + "] ");
974    //                    }
975    //                } else {
976    //                    typedDependenciesStr = "NONE ";
977    //                }
978    //                annotationPrinterStream.println(" --- " + section + ": " + beginIndex + " " + endIndex + "\tANNOTATOR: "
979    //                        + annot.getAnnotator().getAnnotatorID() + "-" + annot.getAnnotator().getFirstName() + "\t" + abbreviatedType
980    //                        + "\t\tPOS: " + ((CCPTokenAnnotation) annot).getPartOfSpeech() + "\tLEMMA: " + ((CCPTokenAnnotation) annot).getLemma()
981    //                        + "\tSTEM: " + ((CCPTokenAnnotation) annot).getStem() + "\tTOKEN_NUM: " + ((CCPTokenAnnotation) annot).getTokenNumber()
982    //                        + "\tTYPED_DEPENDENCIES: " + typedDependenciesStr + coveredText);
983    //            } else if (annot.getType().getName().equals("edu.uchsc.ccp.uima.annotation.syntactic.PhraseAnnotation")) {
984    //                annotationPrinterStream.println(" --- " + section + ": " + beginIndex + " " + endIndex + "\tANNOTATOR: "
985    //                        + annot.getAnnotator().getAnnotatorID() + "-" + annot.getAnnotator().getFirstName() + "\t" + abbreviatedType
986    //                        + "\tPHRASE_TYPE: " + ((CCPPhraseAnnotation) annot).getPhraseType() + "\t" + coveredText);
987    //            } else if (annot.getType().getName().equals("edu.uchsc.ccp.uima.annotation.syntactic.ClauseAnnotation")) {
988    //                annotationPrinterStream.println(" --- " + section + ": " + beginIndex + " " + endIndex + "\tANNOTATOR: "
989    //                        + annot.getAnnotator().getAnnotatorID() + "-" + annot.getAnnotator().getFirstName() + "\t" + abbreviatedType
990    //                        + "\tCLAUSE_TYPE: " + ((CCPClauseAnnotation) annot).getClauseType() + "\t" + coveredText);
991    //            } else { // we assume it is a general Syntactic or SemanticAnnotation
992    //                annotationPrinterStream.println(" --- " + section + ": " + beginIndex + " " + endIndex + "\tANNOTATOR: "
993    //                        + annot.getAnnotator().getAnnotatorID() + "-" + annot.getAnnotator().getFirstName() + "\t" + abbreviatedType
994    //                        + "\tTYPE: " + annot.getClassMention().getMentionName() + "\t" + coveredText);
995    //                UIMA_Util.printCCPClassMention(annot.getClassMention(), annotationPrinterStream, 1);
996    //            }
997    //        }
998    //        annotationPrinterStream.println("==============================================================================");
999    //    }
1000    
1001        /**
1002         * Closes the PrintStream, if one was used by the program.
1003         */
1004        public void collectionProcessComplete(ProcessTrace arg0) throws ResourceProcessException, IOException {
1005    //        if (useEmbeddedXMLFormat) {
1006    //            ((EmbeddedXMLOutput) annotationPrinterUtil).printEmbeddedXMLFooter(annotationPrinterStream);
1007    //        }
1008            if (outputToFile) {
1009                annotationPrinterStream.close();
1010            }
1011            super.collectionProcessComplete(arg0);
1012        }
1013    }
```
